# Supplementary material for: Long-term hepatitis B virus infection of rhesus macaques requires suppression of host immunity
Source: Nat Commun. 2022 May 30;13:2995. doi: 10.1038/s41467-022-30593-0 (PMC9151762; doi:10.1038/s41467-022-30593-0)
Supplement: Supplementary file 1 — Supplementary Information [file 41467_2022_30593_MOESM1_ESM.pdf]

Supplementary Information: Long-term hepatitis B virus infection of rhesus macaques requires suppression of host immunity

Biswas et al.

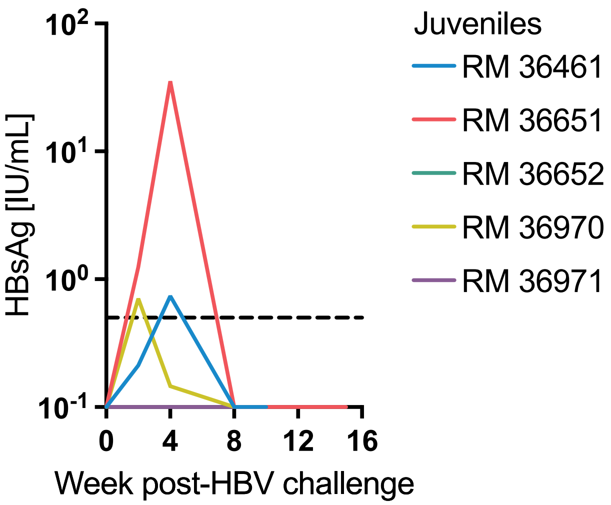

Fig S1. Measurements of HBsAg from the serum of transiently infected RM. Source data are provided as a Source Data file.

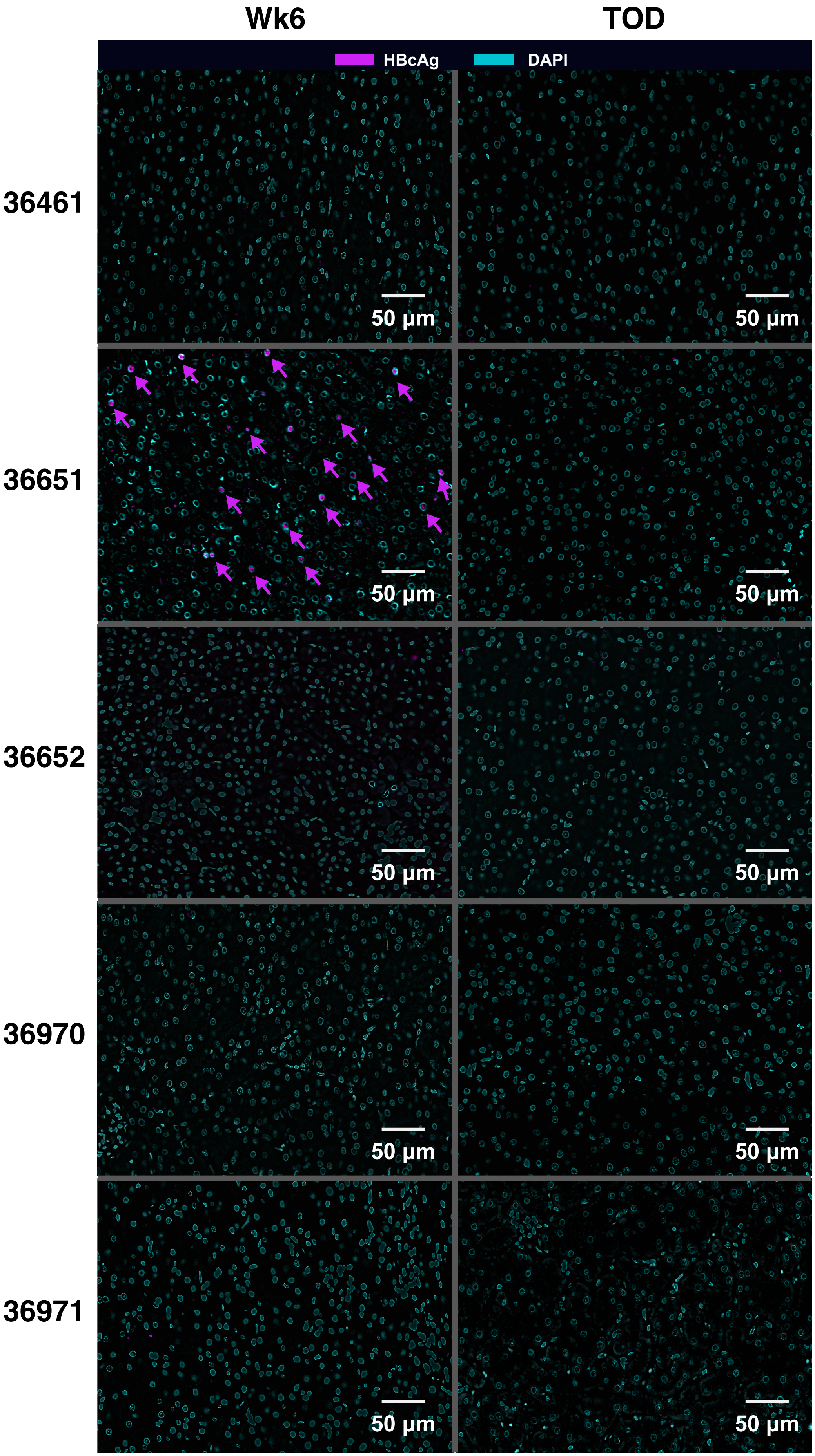

Fig S2. HBcAg staining of liver biopsies from transiently infected RM. Two independent experiments were conducted with similar results. Source data are provided as a Source Data file.

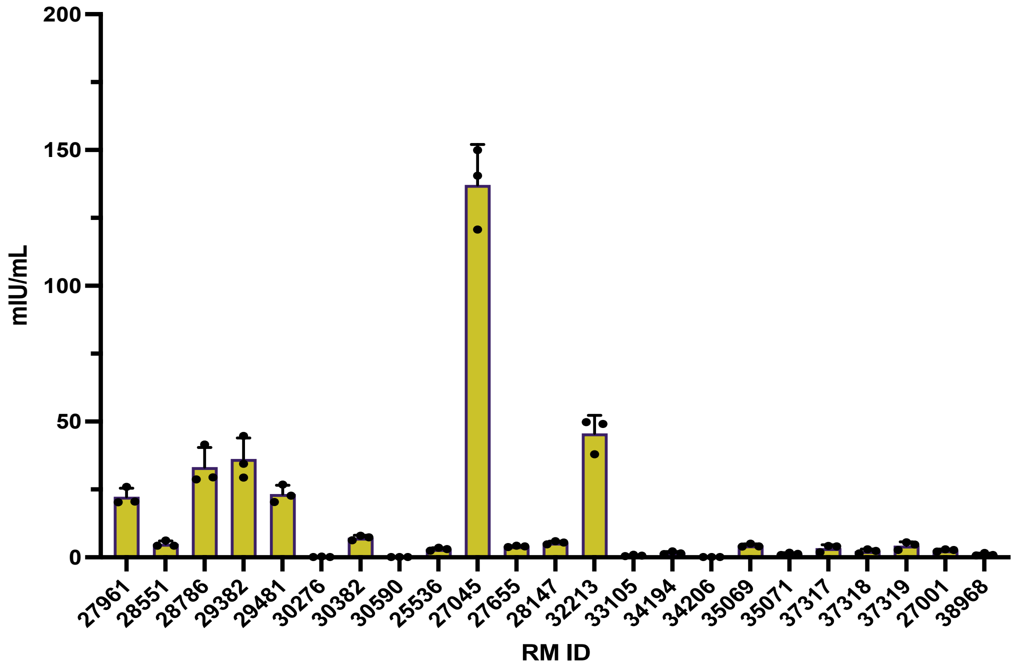

Fig S3. Screening of unvaccinated, HBV-naïve RMs from the ONPRC colony for anti-HBs responses. Data are presented as mean values +/- SD. Source data are provided as a Source Data file.

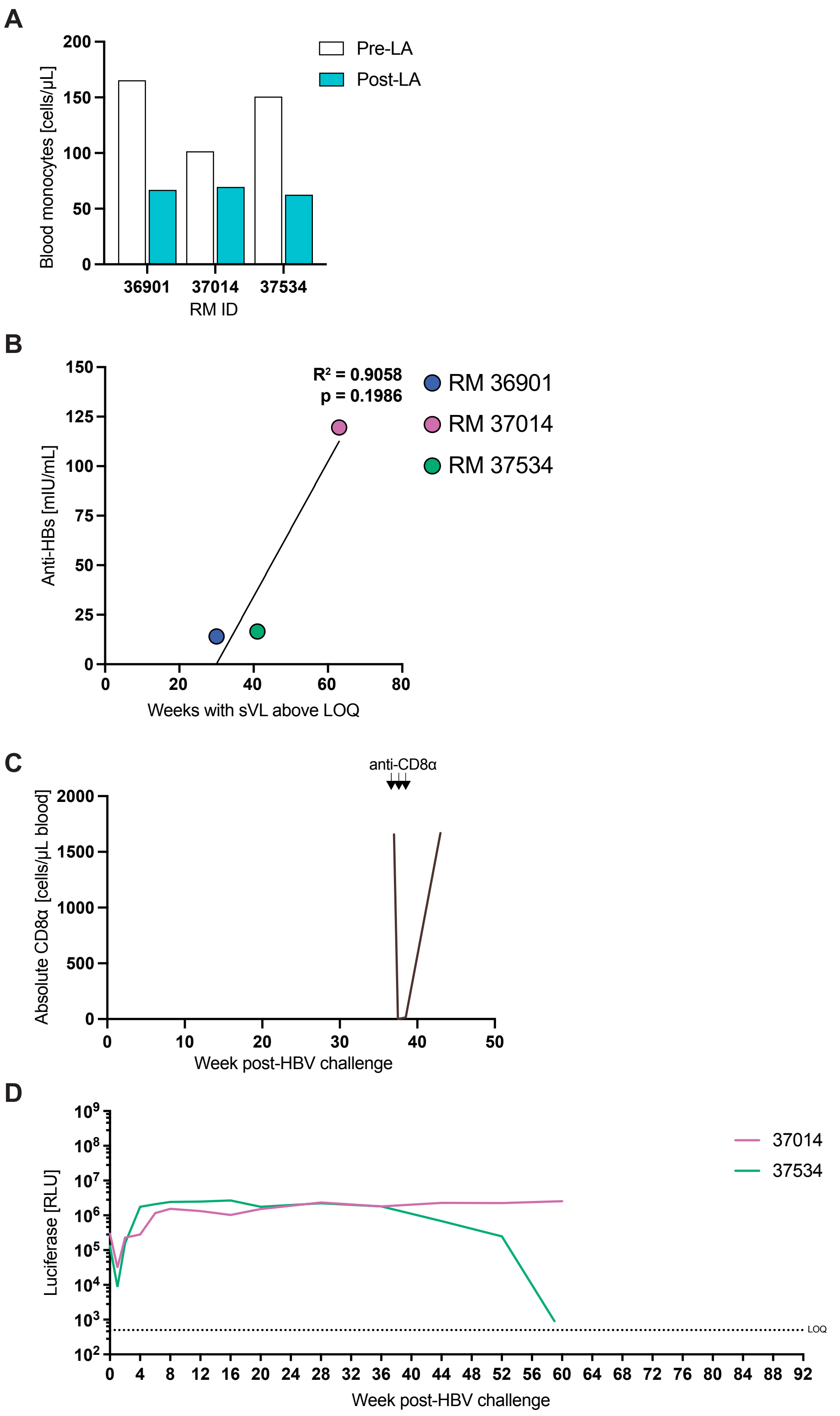

Fig S4. Immune measurements in the blood of RMs. A) Absolute count of monocytes before and after LA administration. B) Correlation between serum anti-HBs levels and the number of weeks with sVL above the limit of quantification. Goodness of fit (R2) and significance (p-value) were determined by simple linear regression. C) Absolute count of CD8α expressing cells before and after administration of a CD8α depleting antibody. D) Neutralization of adenovirus serotype 5 expressing NanoLuc by serum anti-Ad5 antibodies longitudinally in two RM. Source data are provided as a Source Data file.

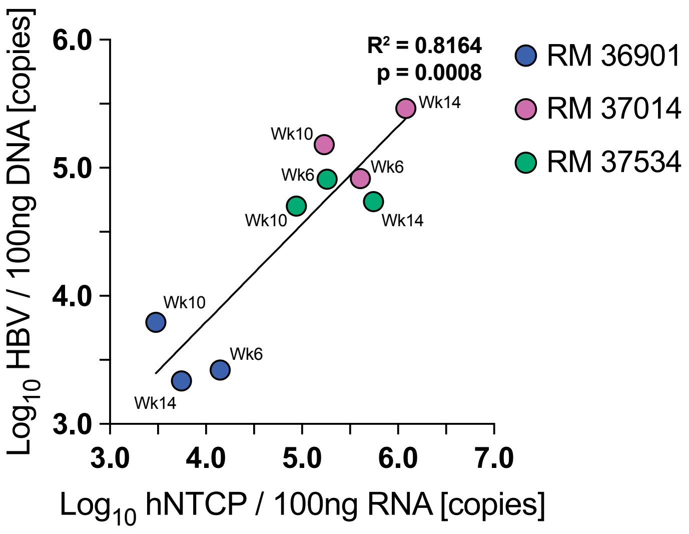

Fig S5. Correlation between HBV DNA and hNTCP RNA in RM livers. Goodness of fit (R2) and significance (p-value) were determined by simple linear regression. Source data are provided as a Source Data file.

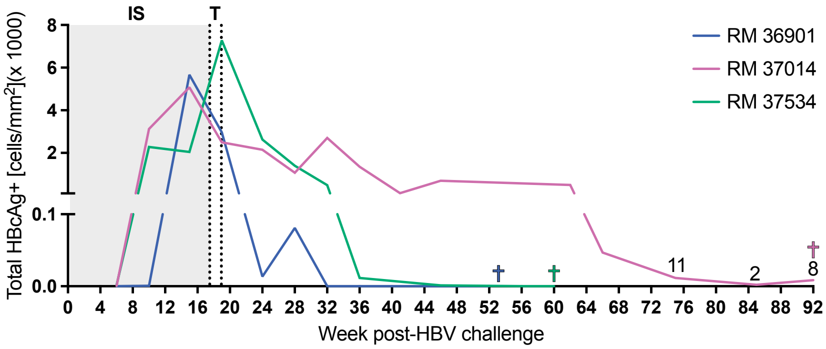

Fig S6. Absolute count of HBcAg+ cells in liver biopsies. Source data are provided as a Source Data file.

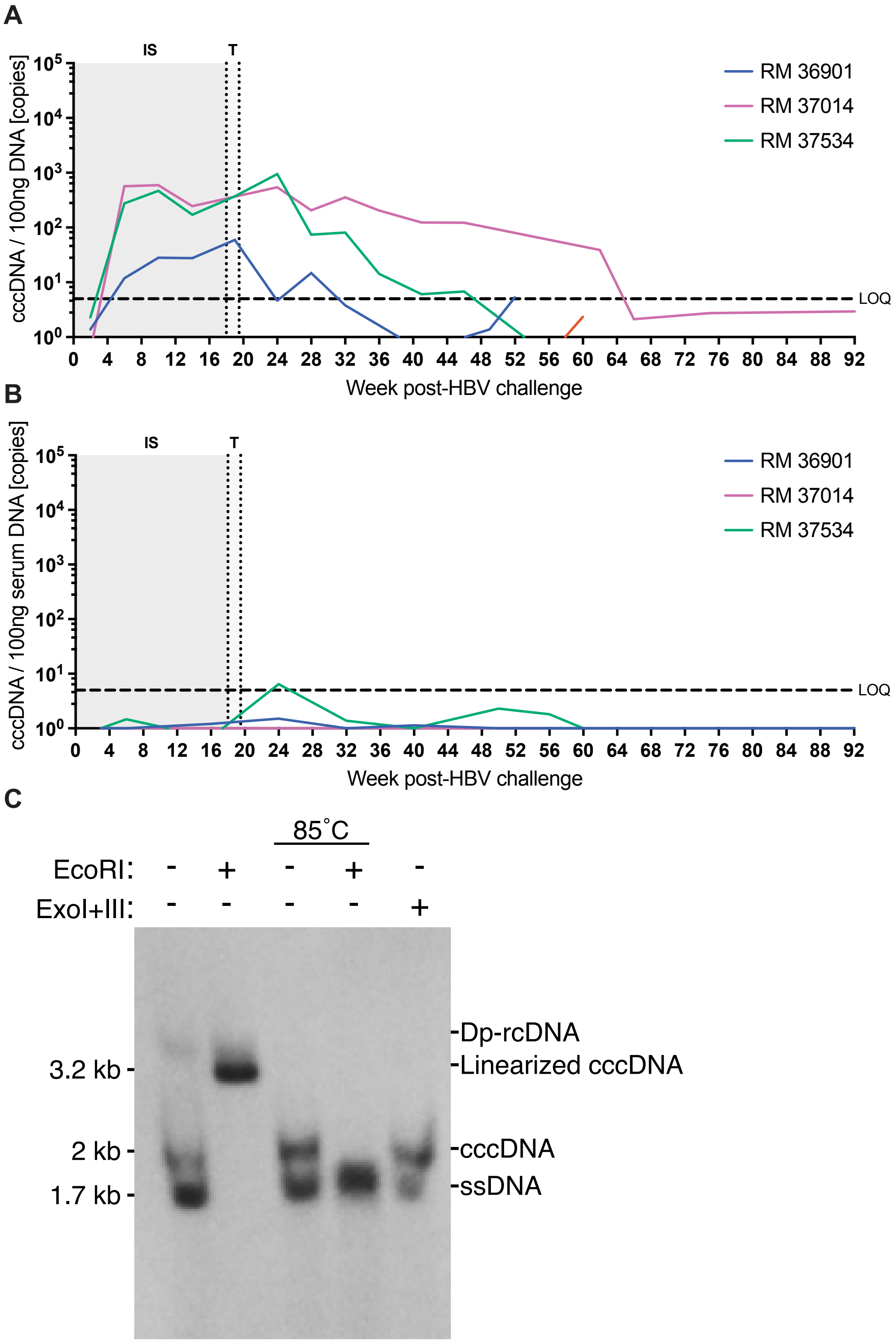

Fig S7. Detection of cccDNA in RM livers. A) Longitudinal quantification of cccDNA copies in the livers of RM. B) Longitudinal quantification of cccDNA copies in the serum of RM. C) Visualization of various forms of HBV DNA, including cccDNA, in the livers of RM. Two independent southern blot experiments were conducted with similar results. Source data are provided as a Source Data file.

	Injected	Dose	Route	DPI																				
				-16	-15	-14	-13	-12	-11	-10	-9	-8	-7	…	-3	…	0	…	5	6	7	8	…	10
RM 36651	Liposomal alendronate (LA)	1 mg/kg	IV																					
	Dexamethasone	1 mg/kg	IM																					
	Ad-hNTCP	1.70E12 particles/kg	IV																					
	HBV	1.00E9 gc	IV																					
	Tacrolimus	0.04 mg/kg	IM																	taper	taper	Stop		
RM 36652	Liposomal alendronate (LA)	1 mg/kg	IV																					
	Dexamethasone	1 mg/kg	IM																					
	Ad-hNTCP	1.70E12 particles/kg	IV																					
	HBV	1.00E9 gc	IV																					
	Tacrolimus	0.04 mg/kg	IM																	taper	taper	Stop		
RM 36970	Liposomal alendronate (LA)	1 mg/kg	IV																					
	Dexamethasone	1 mg/kg	IM																					
	Ad-hNTCP	2.40E12 particles/kg	IV																					
	HBV	1.00E9 gc	IV																					
	Tacrolimus	0.04 mg/kg	IM																	taper	taper	Stop		
RM 36971	Liposomal alendronate (LA)	1 mg/kg	IV																					
	Dexamethasone	1 mg/kg	IM																					
	Ad-hNTCP	2.40E12 particles/kg	IV																					
	HBV	1.00E9 gc	IV																					
	Tacrolimus	0.04 mg/kg	IM																	taper	taper	Stop		
RM 36461	Liposomal alendronate (LA)	1 mg/kg	IV																					
	Dexamethasone	1 mg/kg	IM																					
	Ad-hNTCP	2.40E12 particles/kg	IV																					
	HBV	1.00E9 gc	IV																					
	Tacrolimus	0.04 mg/kg	IM																	taper	taper	Stop		
RM 36314	HDAd-hNTCP	1.00E12 particles/kg	IV																					
	HBV	1.00E9 gc	IV																					
RM 36315	HDAd-hNTCP	1.00E12 particles/kg	IV																					
	AAV8-hNTCP	1.73E13 particles/kg	IV																					
	HBV	1.00E9 gc	IV																					
RM 36355	HDAd-hNTCP	1.00E12 particles/kg	IV																					
	AAV8-hNTCP	2.00E13 particles/kg	IV																					
	HBV	1.00E9 gc	IV																					

Table S1. Procedures on juvenile and infant RMs with transient HBV viremia.

Filled (shaded) cells are marked ■.

Animal	Injected	Dose	Route	-16	-15	-14	…	-10	-9	-8	-7	…	-3	…	0	…	4	…	11	…	22	23	24	25
RM 36901	Belatacept (Nulojix)	20 mg/kg	IV					■					■				■		■					■
RM 36901	Liposomal alendronate	1 mg/kg	IV							■														
RM 36901	Dexamethasone	1 mg/kg	IM							■	■													
RM 36901	Ad-hNTCP	2.40E12 particles/kg	IV								■													
RM 36901	HBV	1.00E9 gc	IV												■									
RM 36901	anti-CD8α	10 mg /kg IM, 5 mg/kg IV	IM/IV																					
RM 36901	Tacrolimus	0.06 mg/kg	IM			■	■	■	■	■	■	■	■	■	■	■	■	■	■	■	■	■	■	■
RM 37014	Belatacept (Nulojix)	20 mg/kg	IV					■					■				■		■					■
RM 37014	Liposomal alendronate	1 mg/kg	IV							■														
RM 37014	Dexamethasone	1 mg/kg	IM							■	■													
RM 37014	Ad-hNTCP	2.40E12 particles/kg	IV								■													
RM 37014	HBV	1.00E9 gc	IV												■									
RM 37014	Tacrolimus	0.08 mg/kg	IM	■	■	■	■	■	■	■	■	■	■	■	■	■	■	■	■	■	■	■	■	■
RM 37534	Belatacept (Nulojix)	20 mg/kg	IV					■					■				■		■					■
RM 37534	Liposomal alendronate	1 mg/kg	IV							■														
RM 37534	Dexamethasone	1 mg/kg	IM							■	■													
RM 37534	Ad-hNTCP	2.40E12 particles/kg	IV								■													
RM 37534	HBV	1.00E9 gc	IV												■									
RM 37534	Tacrolimus	0.08 mg/kg	IM	■	■	■	■	■	■	■	■	■	■	■	■	■	■	■	■	■	■	■	■	■

Animal	Injected	…	39	40	…	53	…	67	…	81	82	83	…	92	93	94	95	…	109	…	120	121	122	123
RM 36901	Belatacept (Nulojix)		■					■		■							■		■					■
RM 36901	Liposomal alendronate																							
RM 36901	Dexamethasone																							
RM 36901	Ad-hNTCP																							
RM 36901	HBV																							
RM 36901	anti-CD8α																							
RM 36901	Tacrolimus	■	■	■	■	■	■	■	■	■	■	■	■	■	■	■	■	■	■	■	■	■	■	■
RM 37014	Belatacept (Nulojix)			■		■		■				■					■		■					■
RM 37014	Liposomal alendronate																							
RM 37014	Dexamethasone																							
RM 37014	Ad-hNTCP																							
RM 37014	HBV																							
RM 37014	Tacrolimus	■	■	■	■	■	■	■	■	■	■	■	■	■	■	■	■	■	■	■	■	■	■	■
RM 37534	Belatacept (Nulojix)			■		■		■				■					■		■					■
RM 37534	Liposomal alendronate																							
RM 37534	Dexamethasone																							
RM 37534	Ad-hNTCP																							
RM 37534	HBV																							
RM 37534	Tacrolimus	■	■	■	■	■	■	■	■	■	■	■	■	■	■	■	■	■	■	■	■	■	■	■

Animal	Injected	125	126	…	137	138	260	263	267	370	371	378	419	420	574	…	584	…	602
RM 36901	Belatacept (Nulojix)																		
RM 36901	Liposomal alendronate																		
RM 36901	Dexamethasone																		
RM 36901	Ad-hNTCP																		
RM 36901	HBV																		
RM 36901	anti-CD8α						IM	IV	IV										
RM 36901	Tacrolimus	taper	taper	taper	Stop														
RM 37014	Belatacept (Nulojix)														■		■		
RM 37014	Liposomal alendronate												■						
RM 37014	Dexamethasone												■	■					
RM 37014	Ad-hNTCP													■					
RM 37014	HBV																		
RM 37014	Tacrolimus	■	taper	taper	taper	Stop									■	■	■	■	Stop
RM 37534	Belatacept (Nulojix)																		
RM 37534	Liposomal alendronate									■									
RM 37534	Dexamethasone									■	■								
RM 37534	Ad-hNTCP										■								
RM 37534	HBV											■							
RM 37534	Tacrolimus	■	taper	taper	taper	Stop													

Table S2. Procedures on juvenile RMs with long-term HBV infection.

Primer name	Sequence
HBV-RCA1	5'- AATCCTCACAATACC -3'
HBV-RCA2	5'- GATGGGATGGGAATA -3'
HBV-RCA3	5'- CCTATGGGAGTGGGC -3'
HBV-RCA4	5'- GCAACGGGGTAAAGG -3'
HBV-RCA5	5'- ATGCAACTTTTTCAC -3'
HBV-RCA6	5'- TCCAAATTCTTTATA -3'
HBV-RCA7	5'- TAGAAGAAGAACTCC -3'
HBV-RCA8	5'- AGAATATGGTGACCC -3'

Table S3. Primers for rolling-circle amplification (RCA) of HBV.
