# Supplementary material for: Long-term hepatitis B virus infection of rhesus macaques requires suppression of host immunity
Source: Nat Commun. 2022 May 30;13:2995. doi: 10.1038/s41467-022-30593-0 (PMC9151762; doi:10.1038/s41467-022-30593-0)
Supplement: Supplementary file 3 — Reporting Summary [file 41467_2022_30593_MOESM3_ESM.pdf]

nature portfolio

Corresponding author(s): Benjamin J. Burwitz

Last updated by author(s): Feb 15, 2022

Reporting Summary

Nature Portfolio wishes to improve the reproducibility of the work that we publish. This form provides structure for consistency and transparency in reporting. For further information on Nature Portfolio policies, see our Editorial Policies and the Editorial Policy Checklist.

Statistics

For all statistical analyses, confirm that the following items are present in the figure legend, table legend, main text, or Methods section.

n/a	Confirmed	
☐	☒	The exact sample size (n) for each experimental group/condition, given as a discrete number and unit of measurement
☐	☒	A statement on whether measurements were taken from distinct samples or whether the same sample was measured repeatedly
☐	☒	The statistical test(s) used AND whether they are one- or two-sided<br>*Only common tests should be described solely by name; describe more complex techniques in the Methods section.*
☒	☐	A description of all covariates tested
☒	☐	A description of any assumptions or corrections, such as tests of normality and adjustment for multiple comparisons
☐	☒	A full description of the statistical parameters including central tendency (e.g. means) or other basic estimates (e.g. regression coefficient) AND variation (e.g. standard deviation) or associated estimates of uncertainty (e.g. confidence intervals)
☐	☒	For null hypothesis testing, the test statistic (e.g. F, t, r) with confidence intervals, effect sizes, degrees of freedom and P value noted<br>*Give P values as exact values whenever suitable.*
☒	☐	For Bayesian analysis, information on the choice of priors and Markov chain Monte Carlo settings
☒	☐	For hierarchical and complex designs, identification of the appropriate level for tests and full reporting of outcomes
☒	☐	Estimates of effect sizes (e.g. Cohen's d, Pearson's r), indicating how they were calculated

Our web collection on statistics for biologists contains articles on many of the points above.

Software and code

Policy information about availability of computer code

Data collection	NanoDrop 2000 software<br>QuantStudio 3 software<br>QuantaSoft Analysis Pro software v1.4<br>AID Classic ELISpot reader software<br>Olympus CellSens Dimension Desktop 1.18
Data analysis	QuantStudio 3 software<br>QuantaSoft Analysis Pro software v1.4<br>FlowJo software v10.8.1<br>Geneious Prime software v2022.0.2<br>Olympus CellSens Dimension Desktop 1.18

For manuscripts utilizing custom algorithms or software that are central to the research but not yet described in published literature, software must be made available to editors and reviewers. We strongly encourage code deposition in a community repository (e.g. GitHub). See the Nature Portfolio guidelines for submitting code & software for further information.

Data

Policy information about availability of data

All manuscripts must include a data availability statement. This statement should provide the following information, where applicable:

- Accession codes, unique identifiers, or web links for publicly available datasets
- A description of any restrictions on data availability
- For clinical datasets or third party data, please ensure that the statement adheres to our policy

Reference sequences used in this study can be accessed through GenBank accession numbers MN645906, NM_003049.4, and U95551.1.
Datasets generated during and/or analyzed during the current study are available on the public data repository FigShare (10.6084/m9.figshare.19529476).

Field-specific reporting

Please select the one below that is the best fit for your research. If you are not sure, read the appropriate sections before making your selection.

[x] Life sciences [ ] Behavioural & social sciences [ ] Ecological, evolutionary & environmental sciences

For a reference copy of the document with all sections, see nature.com/documents/nr-reporting-summary-flat.pdf

Life sciences study design

All studies must disclose on these points even when the disclosure is negative.

Sample size	Given the exploratory nature and the binary readout (long-term infection vs. transient infection) of this study, we utilized small animal groups. Animal numbers for each group were selected based on human data, showing that >80% of infants and young children infected with HBV progress to chronic HBV infection. Using these statistics, we calculated a >99.999% power to detect chronic HBV infection in at least one rhesus macaque.
Data exclusions	No data were excluded.
Replication	In all cases where there was sufficient biological sample (serum, cells, DNA, RNA, etc.) we confirmed results either through repeating identical assays (N=2 assays) or through use of parallel readout assays (ex: cinq-PCR vs T5 exonuclease PCR for cccDNA).
Randomization	Randomization was not used to assign animals to their respective experimental groups. This is due to the fact that no information existed concerning the optimal age, sex, weight, or other demographics that would lead to chronic HBV infection. The only demographic that was used to select all rhesus macaques for all groups was age, as all animals were less than one year of age at the time of assignment and study commencement. This age was chosen to increase chances of chronic HBV infection based on human data.
Blinding	Authors were not blinded to rhesus macaque assignments, as critical clinical decisions are required during longitudinal studies that require the attention of the investigators.

Reporting for specific materials, systems and methods

We require information from authors about some types of materials, experimental systems and methods used in many studies. Here, indicate whether each material, system or method listed is relevant to your study. If you are not sure if a list item applies to your research, read the appropriate section before selecting a response.

Materials & experimental systems

n/a	Involved in the study	
[ ]	[x]	Antibodies
[x]	[ ]	Eukaryotic cell lines
[x]	[ ]	Palaeontology and archaeology
[ ]	[x]	Animals and other organisms
[x]	[ ]	Human research participants
[x]	[ ]	Clinical data
[x]	[ ]	Dual use research of concern

Methods

n/a	Involved in the study	
[x]	[ ]	ChIP-seq
[x]	[ ]	Flow cytometry
[x]	[ ]	MRI-based neuroimaging

Antibodies

Antibodies used	Anti-CD3 clone SP34-2 (BD #558124), anti-CD4 clone OKT4 (Biolegend #317414), anti-CD8 clone SK1 (Biolegend #344714), anti-IFNg clone B27 (BD #554702), anti-TNF clone MAb11 (BD #557068), anti-CD14 clone M5E2 (Biolegend #301838), anti-CD20 clone 2H7 (Biolegend #302314), anti-CD28 clone CD28.2 (BD #556620), anti-CD49d clone 9F10 (BD #555502), anti-Ki67 clone B56 (BD #561284), anti-CD8α clone MT807R1 (Nonhuman Primate Reagent Resource), anti-human IgG Fcγ-specific polyclonal antibodies (Jackson

	ImmunoResearch Laboratories #109-035-098), anti-HBc rabbit polyclonal antibody (Abcam #ab115992), anti-HBs surface antibody clone A10F1 (Biolegend #932302), anti-CD68 clone KP1 (Biocare Medical, Inc. #CM033B), anti-CD163 clone 10D6 (Novocastra/Leica Microsystems Inc. #MA5-11458), HRP-conjugated anti-mouse secondary antibody (GBI Labs #D37-110), anti-CD3 rabbit antibody clone SP7 (Epredia Lab Vision #RM9107S).
Validation	All antibodies were monoclonal, commercially available, and quality controlled by the manufacturer for affinity to cognate human protein by flow cytometry. Cross-reactivity with rhesus macaques was confirmed using the Non-human Primate Reagent Resource Reactivity Database (https://www.nhpreagents.org/ReactivityDatabase) prior to use.

Animals and other organisms

Policy information about studies involving animals; ARRIVE guidelines recommended for reporting animal research

Laboratory animals	Indian-origin Macaca mulatta (rhesus macaque): 36314-F-6 days old, 36315-M-6 days old, 36355-F-5 days old, 36461-M-488 days old, 36651-F-404 days old, 36652-M-488 days old, 36970-M-403 days old, 36971-M-371 days old, 36901-F-388 days old, 37014-F-391 days old, and 37534 -F-187 days old.
Wild animals	No wild animals were used in this study.
Field-collected samples	No field-collected samples were used in this study.
Ethics oversight	Oregon Health and Science University Institutional Animal Care and Use Committee (IACUC)

Note that full information on the approval of the study protocol must also be provided in the manuscript.
